# Implementation of Psychosocial treatments – who’s choice and who’s recovery?

**DOI:** 10.1192/j.eurpsy.2025.44

**Published:** 2025-08-26

**Authors:** T. Wykes

**Affiliations:** Psychology, Institute of Psychiatry, Psychology and Neuroscience, London, United Kingdom

## Abstract

**Abstract:**

Psychosocial interventions play a role in recovery and in the patient’s journey. Recovery is individual and so needs individual responses from the mental health services with individual goals set. Different interventions will be useful at different stages and, of course, they only “work” for some people. Three main strategies are often referred to – reducing symptoms, reducing barriers to recovery, and extending and maintaining recovery to achieve some stable and acceptable (to the patient) optimal level of functioning. Psychosocial intervention strategies are beneficial for each of these, and they are often thought of as independent, but they are inter-related with one type of therapy leading to reductions in the need for other therapies. Even though many of these strategies are included in guidelines, the process of considering which one to start with is a choice. We need to work out how that choice is made.

**Disclosure of Interest:**

None Declared

